# Characterization of the microRNA Expression Profiles in the Goat Kid Liver

**DOI:** 10.3389/fgene.2021.794157

**Published:** 2022-01-10

**Authors:** Xiaodong Zhao, Zhibin Ji, Rong Xuan, Aili Wang, Qing Li, Yilin Zhao, Tianle Chao, Jianmin Wang

**Affiliations:** ^1^ Shandong Provincial Key Laboratory of Animal Biotechnology and Disease Control and Prevention, College of Animal Science and Veterinary Medicine, Shandong Agricultural University, Taian, China; ^2^ Shandong Peninsula Engineering Research Center of Comprehensive Brine Utilization, Weifang University of Science and Technology, Shouguang, China

**Keywords:** microRNA, goat kid, liver, development, ruminant

## Abstract

The liver is the largest digestive gland in goats with an important role in early metabolic function development. MicroRNAs (miRNA) are crucial for regulating the development and metabolism in the goat liver. In the study, we sequenced the miRNAs in the liver tissues of the goat kid to further research their regulation roles in early liver development. The liver tissues were procured at 5-time points from the Laiwu black goats of 1 day (D1), 2 weeks (W2), 4 weeks (W4), 8 weeks (W8), and 12 weeks (W12) after birth, respectively with five goats per time point, for a total of 25 goats. Our study identified 214 differential expression miRNAs, and the expression patterns of 15 randomly selected miRNAs were examined among all five age groups. The Gene ontology annotation results showed that differential expression miRNA (DE miRNA) target genes were significantly enriched in the fatty acid synthase activity, toxin metabolic process, cell surface, and antibiotic metabolic process. The KEGG analysis result was significantly enriched in steroid hormone synthesis and retinol metabolism pathways. Further miRNA-mRNA regulation network analysis reveals 9 differently expressed miRNA with important regulation roles. Overall, the DE miRNAs were mainly involved in liver development, lipid metabolism, toxin related metabolism-related biological process, and pathways. Our results provide new information about the molecular mechanisms and pathways in the goat kid liver development.

## Introduction

The liver is the largest digestive gland and metabolic organ in mammals with an important role in the metabolism of various nutrients as well as harmful substances, and the synthesis of the proteins and digestive juices (Si-Tayeb et al., 2010). The liver, being an important organ for substance metabolism, is endowed with a very rich microvascular network system. It comprises a large number of cells having diverse forms and functions and secrete a variety of enzymes, which ensures the efficient and smooth progression of substance metabolism (Abshagen K et al., 2015). Being an important detoxification organ, the liver metabolizes the toxic and harmful substances that have entered the body (Hoffmann et al., 2012). These substances are then metabolized by the various enzymes in the liver, into products that are less toxic or soluble in bile and are subsequently excreted through the intestines (Andrada and Cortés, 2004). After the birth of animals, there are rapid changes in substance metabolism in the body, as well as in the development and function of tissues and organs (Yin et al., 2013). Although the liver tissues and organs grow rapidly, the various functions mature gradually in due course of time. The regulation of metabolism, as well as liver maturation, is dependent on the exogenous bioactive substances and nutrients. During the primary stage, no nutrients are ingested from the outside world, and the liver does not function perfectly (Reinke and Asher, 2016).

The small molecular nutrients received by the fetus from the mother through the placenta are mainly processed by the liver and subsequently, the macromolecular substances needed for the growth and development of the body are synthesized. During lactation, the nutrients are derived mainly from breast milk, and the liver function undergoes gradual improvement. The nutrients are derived via the digestion of breast milk and are enriched in various ingredients (Prosser, 2021). During the body maturation stage, the nutrients are derived from the food with complex digestive composition, and the liver functions perfectly.

The metabolism of various components from food, including toxic and harmful substances is mainly carried out in the liver (Lima, 1980). Studies have established the liver to be an important organ subjected to epigenetic modification, and nutrient levels capable of affecting the epigenetic modification at RNA and DNA levels (He et al., 2017).

As endogenous non-coding RNAs, miRNAs significantly affect various biological parameters by binding to the target mRNAs and inhibiting the gene expression (Tzur et al., 2009). Numerous studies have accounted for the number of miRNAs for the development, regeneration, and disease management of the liver. For example, miR-122 is specifically expressed in the liver by regulating the liver gene network, which regulates cell cycle, lipid metabolism, inflammation, and tumorigenesis and is essential for liver homeostasis. MiR-122 deficiency is usually associated with diseases such as liver fibrosis, inflammation, steatosis, and cancer (Yin et al., 2016). Members of the miR-181 family are highly expressed in the embryonic liver, encode GATA6 mRNA, and regulate liver organogenesis. Recent studies have shown that both miRNA-150 and miRNA-194 can inhibit the production of hepatic stellate cells (HSCs). (Zaret, 2002).

Detecting the stage-specific or tissue miRNAs and the further exploration of the mechanisms in miRNA dysregulation will serve to enhance our in-depth understanding of the liver function in goat kids, enriching the important molecular regulatory mechanisms along the various developmental stage of the goat kids. Thus, the study expands the miRNA spectrum revealing the possible role of miRNAs in the liver and providing a basis for future research on liver development.

## Materials and Methods

### Ethics Statement

All animal experiments were conducted in accordance with the “Guidelines for Experimental Animals” of the Ministry of Science and Technology and approved by the Laboratory Animal Management and Use Committee of Shandong Agricultural University (Permit Number: SDAUA-2019-021). We strive to reduce the suffering of animals.

### Experimental Sample Collection and RNA Isolation

In this experiment, 25 healthy female Laiwu black goats were selected (Supplementary Table S1). The experimental animals were obtained from the Shandong Fengxiang Animal Husbandry and Seed Industry Technology Co., Ltd. (Laiwu District, Jinan, Shandong Province, China). Taking into consideration, the age and diets of the animals, five ages (1 day, 2, 4, 8, 12 weeks) were selected and each age group had five replicates. The only food source is breast milk for the goat kids, including the colostrum phase (1 day), mid-lactation (2 weeks), and post-lactation (4 weeks). The goat kids were supplemented with a solid concentrate starting on 30 days and were weaned off breast milk at 60 days. The goat kids after 60 days were provided with a solid concentrate starter as the only food source. That is to say, they were provided with breast milk supplemented with a starter (8 weeks) and the starter feeding phase (12 weeks). All the goat kids were collected at an appropriate time to obtain liver tissue for transcriptome analysis. Lastly, all samples were quickly frozen in liquid nitrogen and stored in a refrigerator at -80°C until sequence analysis was performed. To distinguish the samples, the liver samples from the 1-day-old kids were labeled D1-1, D1-2, D1-3, D1-4, and D1-5; the samples from 2-week-old kids were labeled W2-1, W2-2, W2-3, W2-4, and W2-5; the samples from 4-week-old kids were labeled W4-1, W4-2, W4-3, W4-4, and W4-5; the samples from 8-week-old kids were labeled W8-1, W8-2, W8-3, W8-4, and W8-5; the samples from 12-week-old kids were labeled W12-1, W12-2, W12-3, W12-4, and W12-5. Total RNA was extracted from the tissue using Trizol Reagent according to the manufacturer’s instructions (Invitrogen) and genomic DNA was removed using rDNase I RNase-free (TaKara). RNA quality was verified using a 2,100 Bioanalyzer (Agilent Technologies, Santa Clara, CA, United States) and the ND-2000 (NanoDrop Technologies). Only high-quality RNA samples (OD260/280 = 1.8–2.2, OD260/230 ≥ 2.0, RIN≥8, 28S:18S ≥ 1.0, >10 μg) were used to construct sequencing library.

### Small RNA Library Construction and Sequencing

The total RNA was extracted from the liver tissues using TRIzol. 3ug of total RNA was ligated with sequencing adapters with TruseqTM Small RNA sample prep Kit (Illumina, San Diego, CA, United States). Subsequently, cDNA was synthesized by reverse transcription and amplified with 12 PCR cycles to produce libraries. After being quantified by TBS380, deep sequencing was performed by Shanghai Majorbio Bio-Pharm Biotechnology Co., Ltd. (Shanghai, China).

### Data Quality Control in Sequencing

FastQC (Cock et al., 2010) was used to decide the quality control parameters that should be applied. And Fastx was applied for reads filtering. The rules are as follows: The low quality reads were filtered from the data to obtain high-quality reads (reads); the reads were filtered out without 3′ joint but contained 5′ joint, the reads were filtered out without the insert fragment and insert fragment length less than 18 nt and reads containing polyA; the small RNA tags with the frequency of the read less than two were filtered out, and the clean tags sequence of the small RNA was finally obtained.

### Sequencing Data Alignment and miRNA Identification

Low-quality bases (Sanger base quality of <20) of the 3’ end were trimmed using in-house perl scripts, and then the sequencing adapters were removed with the fastx toolkit software (http://hannonlab.cshl.edu/fastx_toolkit/). All identical sequences of sizes ranging from 18 to 32 nt were counted for further analysis. The assembled unique sequences were used for a BLAST search of the Rfam database, version 10.1 (http://rfam.sanger.ac.uk/), to remove non-miRNA sequences (rRNA, tRNA, snoRNA, etc.). The clean reads with the goat reference genome (*Capra hircus* ARS1) were also mapped.

Bowtie (http://bowtie-bio.sourceforge.net/index.shtml) was used to annotate the chromosomal location against the reference genome data. Through a BLAST search of the miRbase, version 21.0 (http://www.mirbase.org/), the perfectly matched sequences were used to count and analyze the known miRNA expression profile. The characteristics of hairpin structure of miRNA precursor can be used to predict novel miRNA. The available software mireap was integrated to predict novel miRNA. At the same time, in-house scripts were used to obtain the identified miRNA base bias on the first position with a certain length and on each position of all identified miRNA. The expression level of each miRNA was calculated according to the transcripts per million reads (TPM) method. Only miRNAs with TPM >0 in at least five samples, and TPM >0 in at least three samples of the same development stage can be used for further differential miRNAs identification.

Significant differently expressed (DE) miRNAs were extracted with |log2FC| >1 and FDR <0.05 by DEseq2 (Love et al., 2014). DEseq2 using a Wald test to calculate the significance testing *p*-value, and adjusted for multiple testing using the procedure of Benjamini and Hochberg.

### MiRNA Expression Profile and PCA Analysis

The miRNA expression level was calculated and normalized for both the known miRNAs and novel miRNAs, using the transcripts per million (TPM). The formula was as follows: TPM = Actual miRNA counts^*^10^6/Total counts of clean tags. The principal component analysis was performed using TPM of all the miRNAs by R package.

### Expression Pattern Analysis of DE miRNAs

To further understand the expression pattern of the DE miRNAs in the liver of the goats at five different developmental stages, the expression pattern of the DE miRNAs was analyzed using the TCseq software (1.14.0) using the c-means method with the parameter dist = “Euclidean,” algo = “cm,” *k* = 5. The expression levels of the DE miRNAs of the goats in five different developmental stages were plotted into line graphs.

### Predicting Target Genes and Analyzing Their Functional Enrichment

To obtain a comprehensive description of the biological characteristics of DE miRNA, all candidate target genes are used in Gene ontology (GO) and Kyoto Encyclopedia of Genes and Genomes (KEGG), with R-package clusterProfiler (He et al., 2012) using Padjust <0.05 as a cutoff (The significant test *p*-value was calculated with Over Representation Analysis, and the procedure of Benjamini and Hochberg has been used for multiple testing).

### Network Analysis

According to the analysis results of GO and KEGG, potential target genes and corresponding miRNAs related to liver development, material metabolism, and immunity were screened out.

According to the GO and KEGG analysis results, potential target genes and corresponding miRNAs related to liver development, material metabolism, and immunity were screened out. Cytoscape (version3.7.2, http://www.cytoscape.org/download.html) was used to draw the regulatory network of differentially expressed miRNAs and target genes. The screening condition for hub miRNAs and hub genes is that the interaction relationship is >10.

### Validation of Quantitative Real-Time PCR

High-throughput sequencing was used to further monitor the differential expression of miRNA in goat liver samples, randomize 15 differently expressed miRNA, and use Real-time LightCycler 480 system (Roche, United States) to detect RT-qPCR. The presence of the primer layer is used to check the design. The qRT-PCR primers used for validation are listed in Supplementary Table S2. Use Primer Prime 5.0 (Premier of Canada) according to the DE-miRNA selection sequence, and check the presence of Mir-X™ -miRNA with the presence of primers according to the application instructions (Clontech).

The reaction volume is 25 μl, including 12.5 μl TB Green Advantage Premix (2X), 0.5 µl ROX Dye (50X), 0.5 µl miRNA-specific primer (10 µM), 0.5 µl mRQ 3 ′ primer (10 µM), 2.0 µl cDNA and 9 µl ddH2O.A 96-well plate was incubated at 95°C for 10 s, 95°C for 5 s, 60°C for 20 s, 95°C for 60 s, 55°C for 30 s and 95°C for 30 s, a total of 40 cycles, with *U6* as an internal reference gene. All reactions were repeated 5 times, and the results were calculated by the 2^−ΔΔCt^ method (Livak and Schmittgen, 2001). Statistical analysis was performed using SPSS Ver 21.0 (IBM Corporation, United States). Correction for multiple tests was determined with one-way ANOVA, Least-significant difference (LSD) and Tukey’s multiple range tests. The bar chart of miRNA expression in different groups was generated by GraphPad Prism 8.0 (GraphPad Software Inc., San Diego, CA, United States) software.

## Results

### Sequencing Data and Comparative Analysis of the Small RNAs

The liver tissue from 25 Laiwu black goats was used for constructing a library, and 300, 492, 036 original reads were obtained from sequencing (Supplementary Table S3). After the assessment of the quality control of the sequencing data, 68, 994, 918 reads of poor quality were filtered out and 231, 497, 118 pure reads were retained. The comparison of the clean reads with that from the Rfam databases, provided the numbers of rRNA, tRNA, snoRNA, snRNA, and repeat reads. Finally, 379,306 ± 25,013, 326,533 ± 14,109, 336,241 ± 26,165, 335,599 ± 26,400, 361,631 ± 30,833 unannotated reads were compared with the genomes at the D1, W2, W4, W8, and W12 groups respectively. (Supplementary Table S4). All data is sent analysis of the unannotated reads with the genome alignment indicated that the reads from all the 25 libraries had genome alignment rates exceeding 89% (Supplementary Table S5). All data have been uploaded to the Gene Expression Omnibus (GEO) database under the accession number PRJNA770352.

### Identification of Known and Novel miRNAs

**FIGURE 1:**
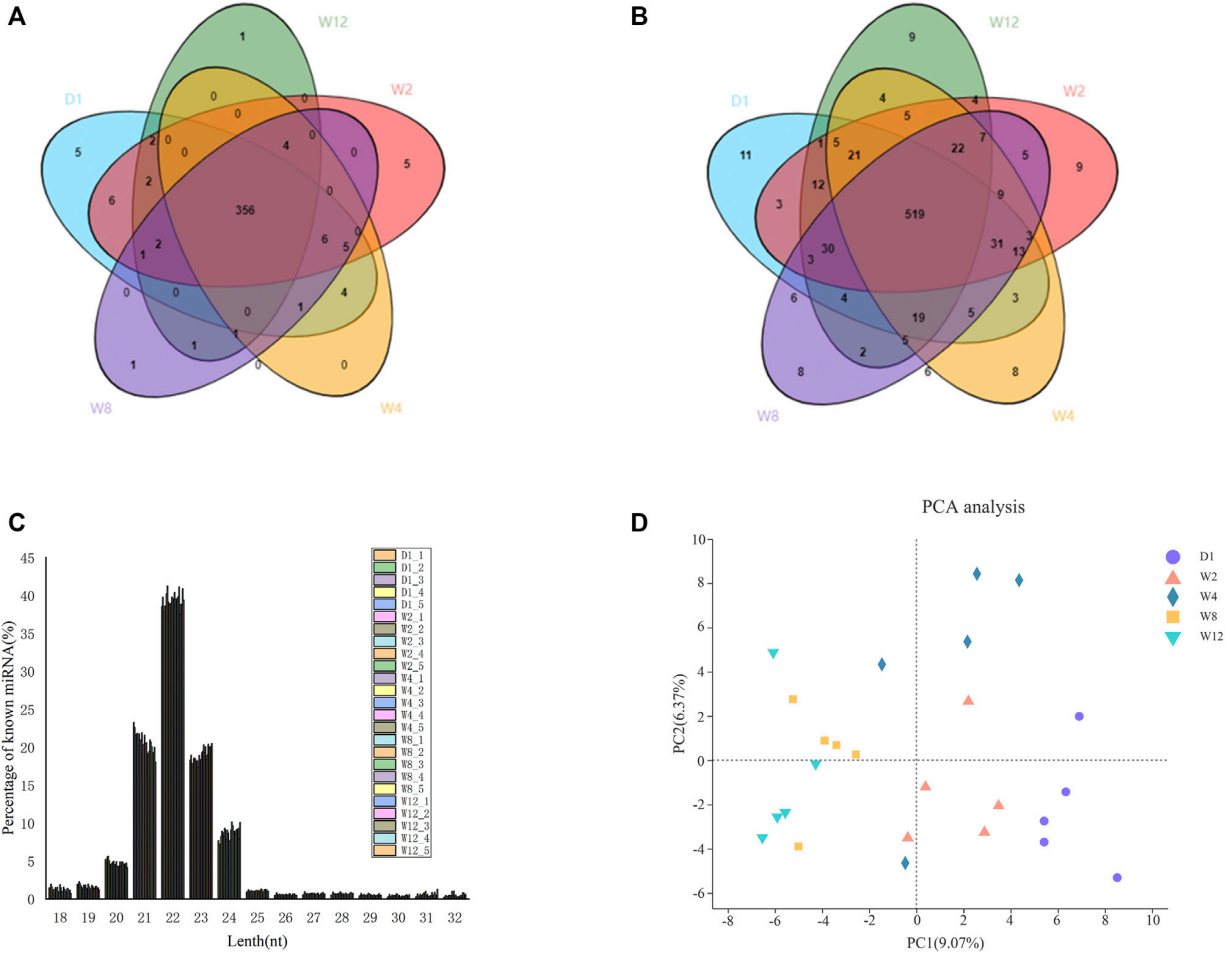
Identification and family analysis of known and novel miRNAs at different developmental stages. **(A)** shows a Venn diagram of known miRNAs at the 25 different stages. **(B)** shows a Venn diagram of novel miRNAs at the 25 different stages. The core circle represents the total number of miRNAs in the 25 libraries. **(C)** shows the distributions of the lengths of known and novel miRNAs. **(D)** represents the principal component analysis.

Screening the 25 libraries provided 1,255 miRNAs, including 463 known miRNAs and 792 novel miRNAs. Among the known miRNA, 395 were expressed in all the 25 libraries (Figure 1A), while among the novel miRNA, 180 were expressed in all 25 libraries (Figure 1B). Analysis of the known and novel miRNA lengths indicated to be concentrated between 20 and 24 nt, with 22 nt miRNA being the most abundant (Figure 1C). To further, determine the miRNA expression patterns in the liver tissues at five different groups, the miRNAs were classified into four groups based on TPM values (Table 1). The number of miRNAs in the D1 of the high expression group was larger than of those in the W2, W4, W8, and W12 groups. In the group of medium expression, the number of miRNAs in the W2 was larger than of those in the other groups. The W12 group, among the low expression and ultra-low expression groups, were higher than other groups, while the D1 was the lowest. According to the expression levels of the top 20 miRNAs in each group, seven of them are highly expressed in each group, as shown in table 2, and they were chi-miR-122, chi-miR-143-3p, chi-miR-21-5p, chi-miR-30a-5p, chi-miR-194, chi-miR-26a-5p, and chi-let-7f-5p.

**TABLE 1:** Transcript expression profiles of the five development stages.

Different expression levels	D1	W2	W4	W8	W12
Highly expressed miRNAs (TPM≥500)	103	94	96	91	91
Mediumly expressed miRNAs (500> TPM≥10)	179	185	178	171	162
Lowly expressed miRNAs	108	108	103	110	116
(10> TPM≥1)					
Ultralowly expressed miRNAs (1> TPM)	63	62	75	72	85

### Differential Expression miRNA Recognition

**FIGURE 2:**
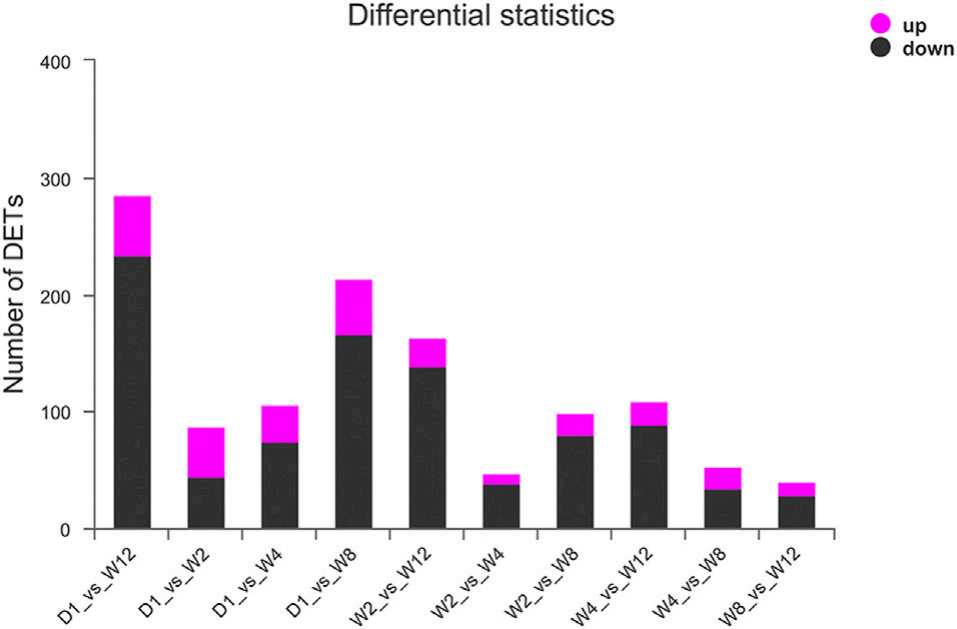
Identification of DE miRNAs and histogram of miRNAs at five different age groups.

The miRNA expression profile of 25 groups from D1, W2, W4, W8, and W12 groups were applied for a Principal Component Analysis (PCA) (Figure 1D). And all miRNA expression data were imported into the DESeq2 software for differentially expression analysis, and corrected by the Wald test and Benjamini-Hochberg for D1 to W2, D1 to W4, D1 to W8, D1 to W12, W2 to W4, W2 to W8, W2 to W12, W4 to W8, W4 to W12, W4 to W8, W4 to W12, and W8 to W12. Here, 754 differentially expressed miRNAs were identified in five groups. We found 51 miRNAs to be upregulated and 66 miRNAs to be downregulated, in comparison between D1 and W2. The novel 1_798 was found to be upregulated by 58.7 times. In comparison between D1 and W4, there was upregulation of 40 miRNAs and downregulation of 99 miRNAs. The novel 1_798 was found to be upregulated by 47.3 times, and the 8_11436 was downregulated by 42.6 times. In comparison D1 and W8, there was the upregulation of 63 miRNAs and the downregulation of 200 miRNAs along with the upregulated novel 1_798 showing the largest change of 53.8 times and the downregulated 29_31074 reached 47.4 times. In the comparison between D1 and W12, there was upregulation of 68 miRNAs and the downregulation of 268 miRNAs; moreover, 13 of these miRNAs showed more than 20 times difference. The expression level of novel 1_798 was upregulated by 79.5 times, and that of novel 29_31074 was downregulated by 48.7 times. Comparing W2, W4 revealed an upregulation of 23 miRNAs up-regulated and downregulation of 60 miRNAs. chi-miR-497-3p downregulation was 74.6 times. W2 in comparison to W8 showed 34 miRNAs to be upregulated and 98 miRNAs to be downregulated. Besides, 25_28017 was found to be upregulated by 17.6 times, and chi-miR-497-3p was downregulated by 69.4 times. W2, compared to W12, showed an upregulation of 35 miRNAs and downregulation of 169 miRNAs with 26_29338 and 4_4907 being upregulated by 22 times, and 9_12315 being downregulated by 67.1 times. W4 in comparison to W8 showed an upregulation of 27 miRNAs and downregulation of 48 miRNAs; 21_24338 was upregulated by 53.4 times. In W4, 33 miRNAs were found to be upregulated and 126 miRNAs were downregulated compared to W12, and the chi-miR-497-3p was upregulated by 56.7 times, and 9_12315 was downregulated by 69.8 times. Compared to W12, W8 showed an upregulation of 29 miRNAs and downregulation of 45 miRNAs; also, the chi-miR-497-3p was upregulated by 52.7 times, and 20_24084 was downregulated by 22.6 times. Figure 2 and Supplementary Table S6 provides the detailed differential expression.

### Expression Analysis of the Different miRNA Between the Groups

**FIGURE 3:**
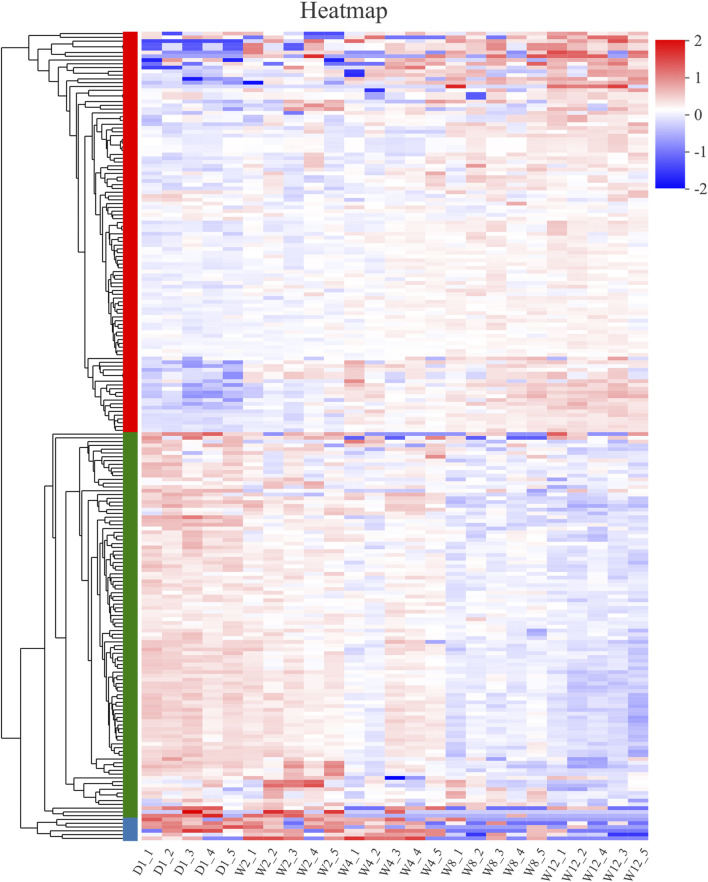
Clustering pattern of DE miRNAs between different groups. D1-1, D1-2, D1-3, D1-4, D1-5 were the samples for 1-day-old kids, W2-1, W2-2, W2-3, W2-4, W2-5 were the samples for 2-week-old goats, W4-1, W4-2, W4-3, W4-4, W4-5 were the samples for 4-week-old goats, W8-1, W8-2, W8-3, W8-4, W8-5 were the samples for 8-week-old goats, W12-1, W12-2, W12-3, W12-4, W12-5 were the samples for 12-week-old goats.

The log2 (FC) > 2 and Padjust< 0.05 threshold were analyzed to identify the different expressions of 214 different miRNA between the groups. The expression levels of these 214 differential miRNAs in each group were mapped and the cluster was analyzed (Figure 3).

### MiRNAs Clustered Into the Same Category had Similar Expression Patterns

**FIGURE 4:**
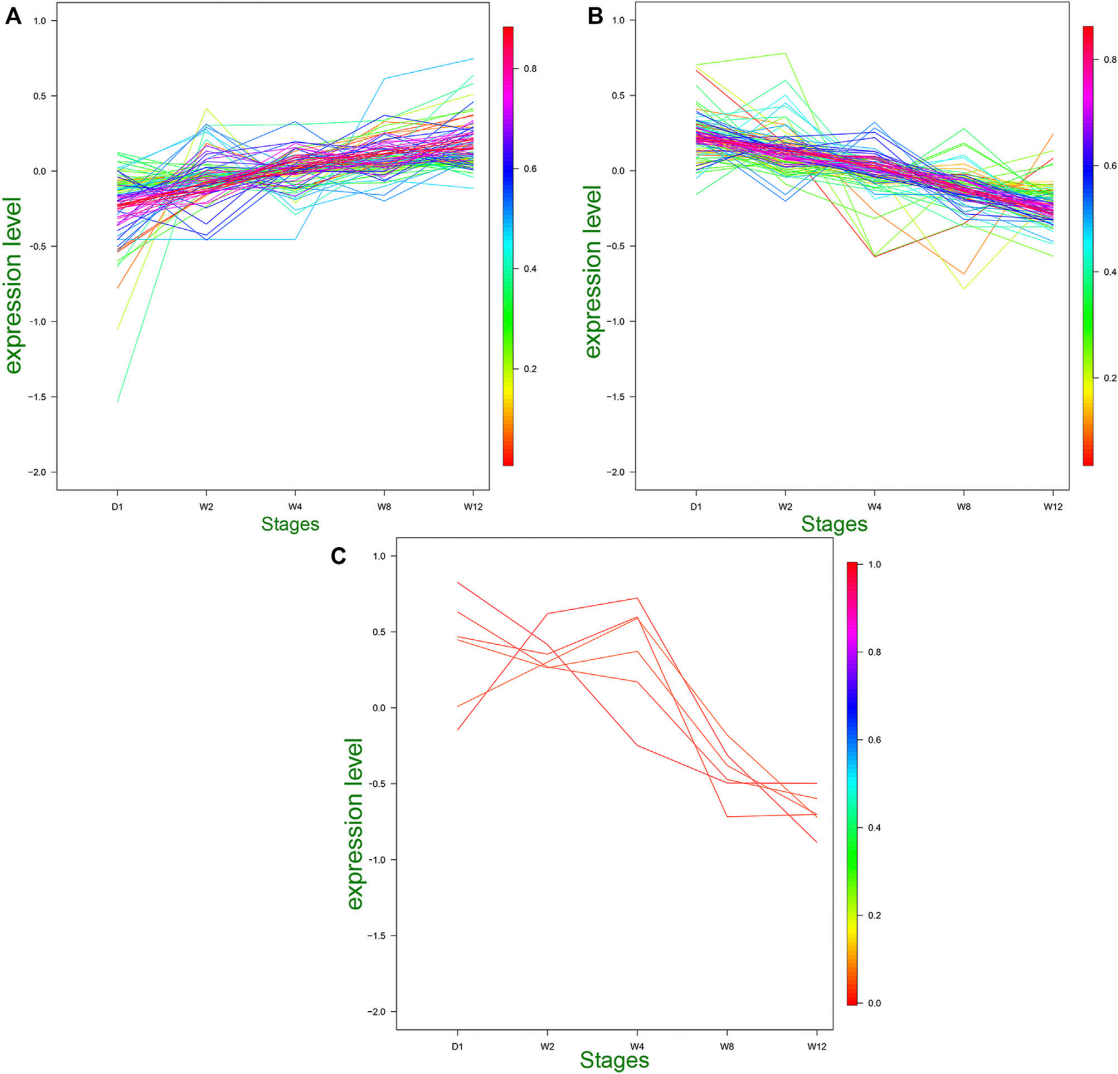
Trends of medium and above expression of DE miRNAs. **(A)** Cluster 1 shows the DE miRNAs that gradually increased in D1, W2, W4, W8, and W12. **(B)** Cluster 2 shows the DE miRNAs that gradually decreased in D1, W2, W4, W8, and W12. **(C)** Cluster 3 shows the DE miRNAs that gradually increased in D1, W2, W4 and gradually decreased in W8, W12.

Based on the heat map clustering, the miRNAs were clustered into three categories: the first category with 106 differentiated miRNAs, the second category with 102 differentiated miRNAs, and the third category with six differentiated miRNAs (Figure 4). The first cluster indicated an upregulation of 106 miRNAs in the W8 and W12 groups, and their expression levels were upregulated with the aging of the Laiwu black goats (Figure 4A). In the second cluster indicated the high expression of miRNAs in group D1, and their expression levels were downregulated with age (Figure 4B). The second cluster of miRNAs may be involved in regulating the transcription level and stress response. The third cluster indicated upregulation of six differential miRNAs in the D1, W2, and W4 groups, and downregulation in the W8 and W12 groups (Figure 4C). Therefore, the transcriptional level regulation was suggested to be involved, and therefore, these differential miRNAs be related to the dietary changes. The target genes of these three clusters were analyzed by GO and KEGG enrichment.

### Prediction and Enrichment Analysis of the miRNA Target Genes

#### GO Annotation Analysis

**FIGURE 5:**
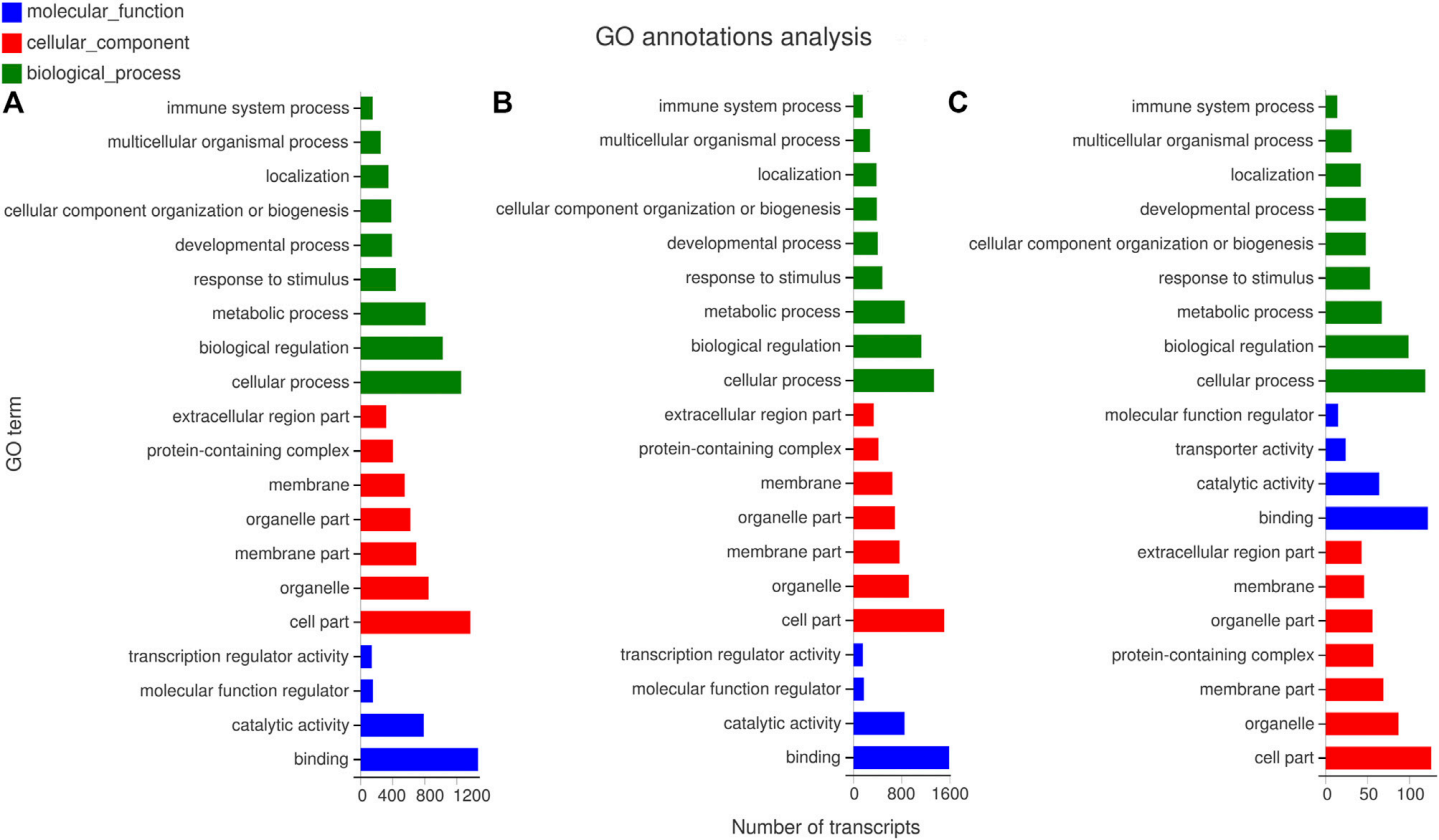
GO enrichment analysis of miRNA predicted target genes. **(A)** The GO enrichment analysis of target genes predicted by miRNAs with different expression patterns in cluster 1. **(B)** The GO enrichment analysis of target genes predicted by miRNAs with different expression patterns in cluster 2. **(C)** The GO enrichment analysis of target genes predicted by miRNAs with different expression patterns in cluster 3.

The GO was analyzed on the target genes of DE miRNAs to clarify the main functions and regulatory mechanisms involved in the DE miRNAs (Figure 5 and Supplementary Table S7). The GO of the first cluster identified 29,080 target genes to be enriched into 1,477 GO terms, including 95 cell component terms, 272 molecular function terms, 1,110 biological process terms. Among them, four4 cell component terms (CC), 23 molecular function terms (MF), and 51 biological process terms (BP) significantly enriched. The cell surface (GO:0009986), arachidonic acid epoxygenase activity (GO:0008392), and cellular and antimicrobial process (GO:0016999) demonstrated the highest enrichment significance in CC, MF, and BP terms, respectively. extracellular space (GO:0005615), binding (GO:0005488), arachidonic acid epoxygenase activity (GO:0008392) demonstrated the largest number of enriched target genes in CC, MF, and BP terms, respectively (Figure 5A).

In the second cluster, 38,360 differential target genes enriched in 1,457 GO terms, including 112, 309, and 1,036 are in CC, MF, and BP terms, respectively. Furthermore, 2, 18, and 24 terms demonstrated the highest enrichment significance in CC, MF, and BP terms among them, respectively. In CC term, the extracellular matrix (GO:0031012) showed the largest number of enriched target genes, and the collagen-containing extracellular matrix (GO:0062023) showed the highest enrichment significance. In MF term, the largest number of enrichment target genes was for tetrapyrrole binding (GO:0046906), and the highest enrichment significance was the fatty acid synthase activity (GO:0004312). In BP term, the largest number of enriched target genes was the monocarboxylic acid metabolic process (GO:0032787), and the toxin metabolic process (GO:0009404) was the highest enrichment significance (Figure 5B).

The GO enrichment was analyzed with the target genes of the third cluster, where 215 differential target genes were enriched into 700 GO terms, which included 81, 102, and 517 are in CC, MF, and BP terms. Furthermore, there were 2, 9, and 20 terms that significantly enriched in CC, BP, MF terms, respectively. there are no significantly enriched terms in MF. In CC term, the receptor complex (GO:0043235) possess the highest enrichment significance and the largest number of enrichment target genes. In BP terms, it significantly enriched in regulation of platelet-derived growth factor production (GO:0090361), biological regulation (GO:0065007), DNA endoreduplication (GO:0042023), trophectodermal cell proliferation (GO:0001834), pre-mRNA catabolic process (GO:1990261), Cajal body organization (GO:0030576), cell cycle DNA replication (GO:0044786), positive regulation of transcription involved in G1/S transition of mitotic cell cycle (GO:0071931), negative regulation of motor neuron apoptotic process (GO:2000672), regulation of motor neuron apoptotic process (GO:2000671), regulation of platelet-derived growth factor production (GO:0090361). These terms also have the largest number of target genes (Figure 5C).

### KEGG Enrichment Analysis

**FIGURE 6:**
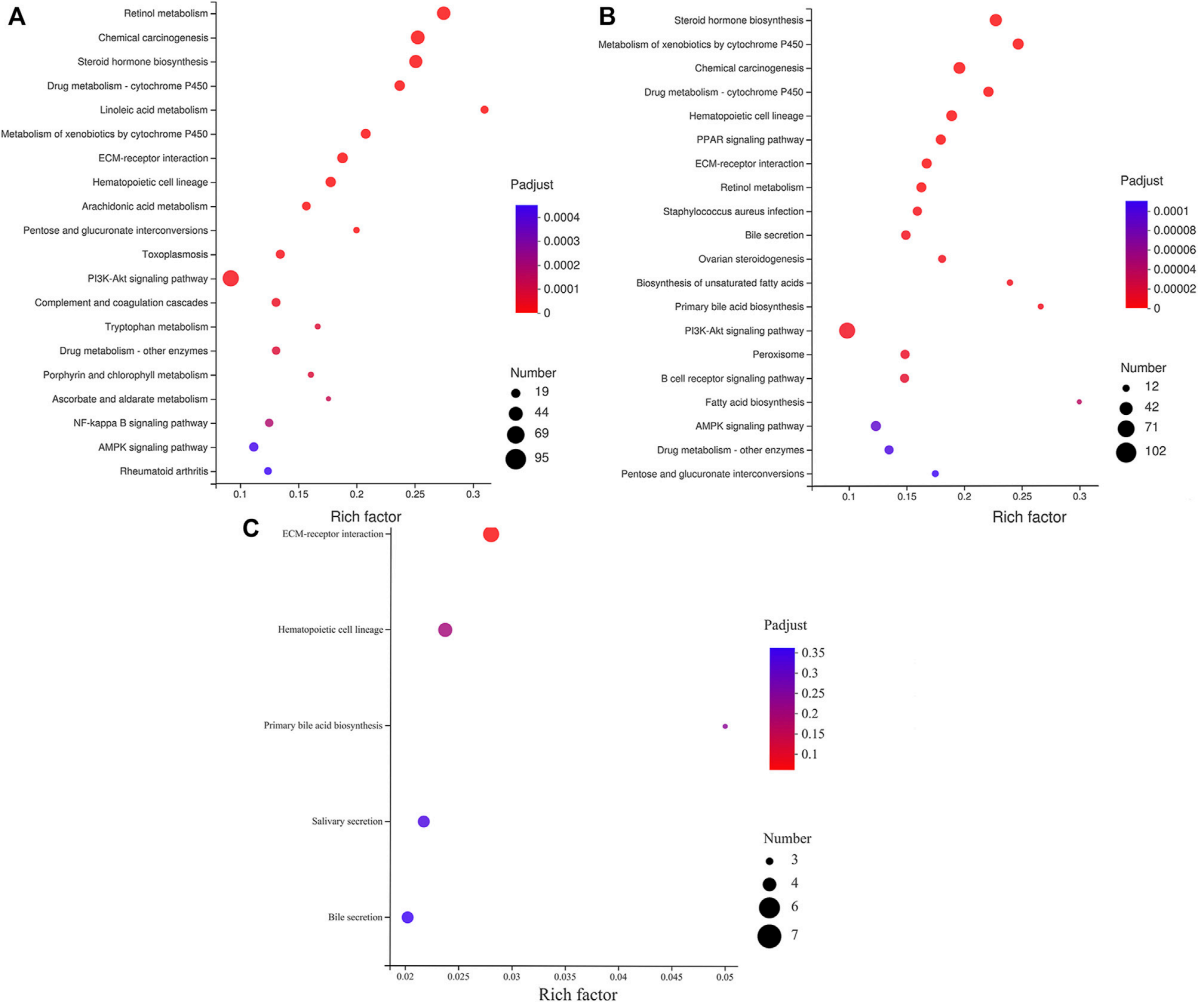
KEGG enrichment analysis of miRNA predicted target genes. **(A)** The KEGG enrichment analysis of target genes predicted by miRNAs with different expression patterns in cluster 1. **(B)** The KEGG enrichment analysis of target genes predicted by miRNAs with different expression patterns in cluster 2. **(C)** The KEGG enrichment analysis of target genes predicted by miRNAs with different expression patterns in cluster 3.

The analysis of the KEGG Pathway enrichment for the target genes of DE miRNAs was performed (Figure 6 and Supplementary Table S6). The results show that 1751 target genes of the first cluster are enriched in 321 pathways, 30 of which have reached a significant enriched, the most significant enrichment is retinol metabolism, chemical carcinogenesis, steroid hormone biosynthesis, drug metabolism - cytochrome P450, linoleic acid metabolism, and so on. Moreover, the largest number of enriched target genes is the PI3K-Akt signaling pathway (Figure 6A). 1881 target genes of the second cluster are enriched in 316 pathways, 36 of which are significantly enriched, the most significant enrichment is steroid hormone biosynthesis, metabolism of xenobiotics by cytochrome P450, chemical carcinogenesis, drug metabolism - cytochrome P450, hematopoietic cell lineage, and so on. Furthermore, the pathway with the largest number of enriched genes is pathways in cancer (Figure 6B). In the third cluster, there were 31,901 target genes enriched in 182 metabolic pathways without significant difference (Figure 6C).

### Regulatory Networks of the miRNAs and Their Target Genes

**FIGURE 7:**
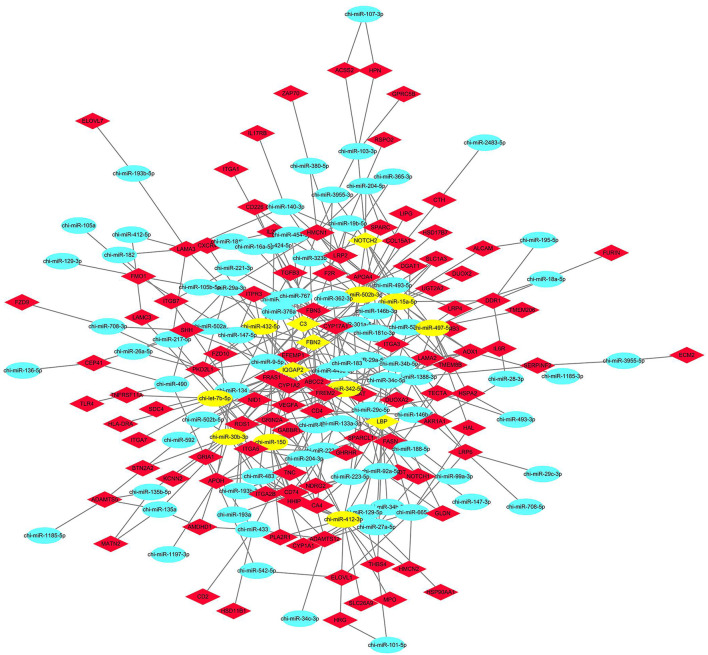
Protein-protein interaction network and network analysis for core genes and miRNAs.

According to the results of the GO and KEGG target gene annotation, a target regulatory network was constructed using a total of 107 genes, and 78 DE miRNAs were selected from seven GO terms and two KEGG pathways (Figure 7). According to the degree of interaction, the network contained nine core miRNA and five core genes. The top nine miRNAs are chi-let-7b-5p, chi-miR-15a-5p, chi-miR-497-5p, and chi-miR-150, chi-miR-342-5p, chi-miR-30b-3p; chi-miR-432-5p, chi-miR-412-3p, and chi-miR-502b-3p. The top five genes are IQGAP2, LBP, NOTCH2, C3, and FBN2. Notably, chi-let-7b-5p and chi-miR-432-5p are all target FBN2, chi-miR-497-5p and chi-miR-432-5p are all target NOTCH2, chi-miR-432-5p is also target IQGAP2, chi-miR-502b-3p target C3, chi-miR-15a-5p has the largest number of target genes.

### Experimental Validation

**FIGURE 8:**
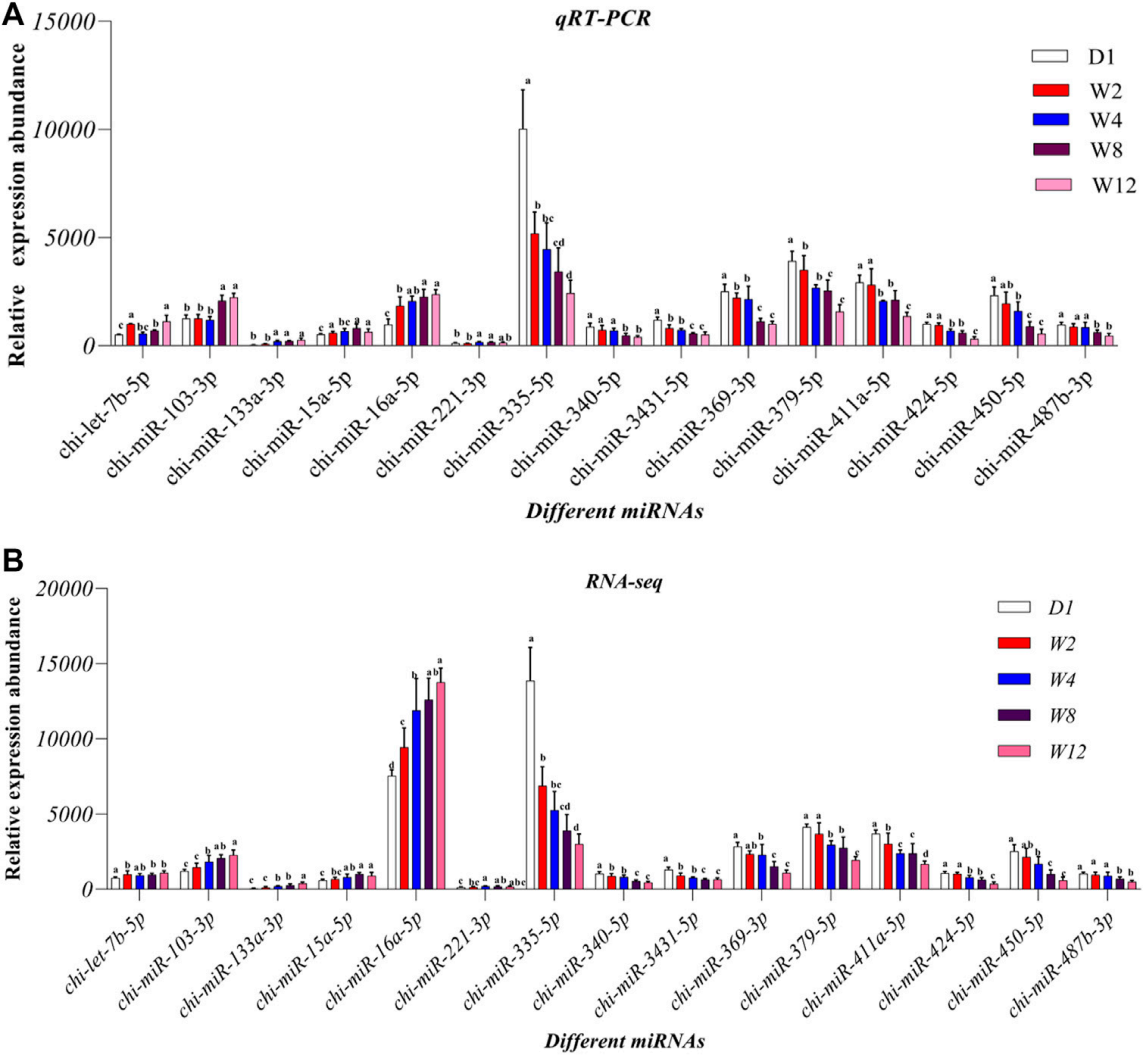
Comparison of relative expression of DE miRNAs between RNA-Seq and qRT-PCR results. **(A)** the results of qRT-PCR, **(B)** the results of RNA-seq.

To check the accuracy of the sequencing and analysis results 15 DE miRNAs were selected for RT-qPCR validation (Figure 8). The results of most selected miRNA qRT-PCR are consistent with the results of the RNA sequence.

## Discussion

This study sequenced the miRNA in the liver of goat kids at five groups. A number of studies have shown that the early nutritional planning of feed for postpartum lambs affects the metabolism, growth, development, and health of the entire life (Greenwood et al., 2004; Galvani et al., 2014). After birth, animals must adapt to new conditions and metabolic pathways (Baldwin et al., 2004; van den Borne et al., 2007). So nutritional supply can regulate these adaptive changes, which means that the nutritional status early in life can have a long-term impact on health in the post-weaning phase (Santos et al., 2018). As the largest digestive gland in the animal body, the liver participates in the metabolism of amino acids, sugars, fats, and other substances in the body, and may have key functions in the early developmental stage. miRNAs can potentially regulate every aspect of cellular activity, from differentiation and proliferation to apoptosis, and also modulate a large range of physiological processes from developmental timing to organogenesis (Kiss, 2002; Nelson et al., 2003; Ambros, 2004; Bartel, 2004; Voinnet, 2005; Taganov et al., 2007). MiRNAs also modulate a diverse spectrum of liver functions with developmental, physiological, and clinical implications (Girard et al., 2008; Liu et al., 2017). These studies indicate that miRNAs play important roles in liver development and functional maintenance. However, currently, reports on miRNA function in the liver of goat kids are rare. To the best of our knowledge, this is the first miRNA sequencing and expression profiling study on the liver of goat kids in five different development stages.

At present, the functional studies of the liver miRNAs were mainly focused on humans and rats. Extensive studies have been performed in studying the biological roles of miRNAs. The miRNA122 accounts for more than half of starlings expressed in the liver and has a variety of physiological and pathological functions, including improving hepatitis virus replication, regulating lipid metabolism, and inhibiting liver cancer (Otsuka et al., 2017). It is reported that the miRNAs are closely linked to liver regeneration, block or stimulate the miRNA pathway in liver regeneration might serve as a novel therapeutic strategy in regeneration-related liver management in the future (Yi et al., 2016). MiRNA is involved in almost all aspects of biology, including cell differentiation, metabolism, proliferation, and apoptosis during tumor formation, as well as the stability of mobile genetic elements and viral infections (Kim et al., 2009). A number of genes in humans are regulated by miRNAs (He and Hannon, 2004). The processes of many organ developments and differentiation are found to be intensely regulated by miRNAs. The miRNAs overexpress or under-express affecting the expression of the gene, and likewise, the expression of the related pathways causing lesions. MiRNA is a potential biomarker for treatment response monitoring and diagnosis, and an imminent new treatment target (Jahn et al., 2020). From clinical trials to laboratory tests, doctors should expect stable miRNA formulas. For example, miRNA is a powerful diagnostic marker for alcoholic liver disease (ALD) patients (Miranda et al., 2010). The serum levels of miR-192 and miR-30a are related to the diagnosis of ALD (Momen-Heravi et al., 2015).

In our study, seven of the top 20 miRNAs are highly expressed in each group, they were chi-miR-122, chi-miR-143-3p, chi-miR-21-5p, chi-miR-30a-5p, chi-miR-194, chi-miR-26a-5p, and chi-let-7f-5p, The expression of these miRNA in the five developmental stages is no different, indicating that these miRNA play an important and stable role in the liver development of goat kids. The expression level of miR-122 is the highest in goat kids at all stages. It is reported that the lack of severe acute toxicity in mice with a congenital absence of miR-122 is maybe a consequence of their livers that never develop a fully differentiated hepatocyte expression pattern and retain expression of genes typically restricted to development (Valdmanis et al., 2016). MiR-122 modulates lipid metabolism and suppresses tumor formation, and sequestration by Hepatitis C virus (HCV) may influence virus pathogenesis (Kunden et al., 2020). MiR-122 modulates glutamine (Gln) metabolism both *in vitro* and *in vivo*, implicating the therapeutic potential of miR-122 in hepatocellular carcinoma (HCC) that exhibit relatively high GLS levels (Sengupta et al., 2020). These studies indicate that miR-122 may be involved in the process of cell proliferation, lipid metabolism, and disease occurrence in the liver of goat kids. During early chondrogenic differentiation, the level of miR-143-3p was decreased, and miR-143-3p could regulate the differentiation process by targeting BMPR2 in BMSCs (Tian et al., 2018).

Lv et, al. found that miR-21-5p may reduce the apoptosis and inflammation in spinal cord tissues of rats through the PI3K/AKT pathway (Lv et al., 2020). However, the function of miR-26a in the liver of goat has not been reported. It is reported that there is an uncharacterized mechanism of biochemical resistance to hormone therapies orchestrated by the miR-30a-5p/CLCF1 axis to mediate sorafenib resistance and aerobic glycolysis in HCC, and targeting the miR-30a-5p/CLCF1 axis may contribute for therapeutic HCC sorafenib resistance patients (Zhang et al., 2020). MiR-194 revealed significant regional differences in the development of bovine gastrointestinal tract during early life, and they maybe be potential regulators of gut tissue cell proliferation and differentiation (Liang et al., 2014). Studies show that miR-194 inhibits liver cancer stem cell expansion by regulating RAC1 pathway (Ran et al., 2019). Studies have shown that miR-194 inhibits the expression of PTBP1 by binding to the 3′-UTR of PTBP1 mRNA, and induces the reduction of CCND3 levels and the growth of liver cancer cells; in addition, the alignment with the miR-194/PTBP1/CCND3 axis can be used as a new strategy for the treatment of human HCC is used (Kang et al., 2019). So we hypothesize the stable expression of chi-miR-194 in liver of goat kids is also related to the growth and development of liver cells, which need further study. MiR-26a-5p may regulate milk fat synthesis in ruminants by targeting INSIG1 (Wang et al., 2016). Using exosomes to deliver miR-26a may treat complications associated with chronic kidney disease (Wang et al., 2019). By regulating the PTEN/PI3K/AKT signaling pathway, MiR-26a-5p can protect cardiomyocytes from I/R damage and provide an opportunity to treat myocardial I/R damage (Xing et al., 2020). Therefore, we speculate that chi-miR-26a may regulate related signal pathways through targeting and regulate liver lipid metabolism in young goats. As a potential inhibitor of Helper T cells (Th17) differentiation in the pathogenesis of MS, let-7f-5p targets STAT3 and serves as a new therapeutic target (Li et al., 2019). Up-regulation of let-7f-5p has been found to ameliorate inflammation by modulating NLRP3 *in vivo*. (Chen H et al., 2019; Tan et al., 2019). These miRNAs may have crucial roles in liver development in goat kids and are not associated with the differential regulation of the transcription levels at different developmental stages. Their expression level changing may lead to cell proliferation, apoptosis, pathological or physiological changes triggering the disease. The analysis of DE miRNA showed that the number of DE miRNAs compared between D1 and W12, W2 and W12, D1 and W8 groups were greater than 100. Besides, there was a significant down-regulation of DE miRNAs by more than 80%, compared to only 16 DE miRNAs between the W8 and W12 groups. From birth to weaning, the miRNA was found to be expressed stably in the liver of goats at the weaning stage suggesting that miRNAs stabilize the liver function in goats after gradual weaning. This indicates that DE miRNAs may play regulatory factors in the liver development of goat kids.

To study the function of DE miRNA with different expression profiles, the expression patterns of DE miRNAs were analyzed identifying three clusters. The expression profile of DE miRNA was correlated with the developmental stages of goats. We found that the target genes in the three clusters were enriched by KEGG pathways. The DE miRNAs were found to express at low levels at birth and high levels in the liver of weaned goats (Cluster 1) and their target genes were significantly enriched in Metabolism and Human Diseases. Such as retinol metabolism, chemical carcinogenesis, steroid hormone biosynthesis, drug metabolism - cytochrome P450, linoleic acid metabolism, and the largest number of enriched target genes is PI3K-Akt signaling pathway. It is reported that colostrum with a higher concentration of vitamin A may be better when feeding newborn lambs, (Buranakarl et al., 2021). We speculate that due to changes in the diet of goats after birth, the content of vitamin A in the diet changes from lactation to weaning, and the expression of genes related to retinol metabolism increases. Follicle-stimulating hormone promotes retinol uptake and its conversion to retinoic acid by regulating the pathways of retinol uptake and metabolism in the mouse ovaries (Swanson et al., 2000). Buffering agent via insulin-mediated activation of PI3K/AKT signaling pathway to regulate lipid metabolism in lactating goats (Li et al., 2018). Therefore, we speculate that there may be some factors in the liver of young goats to promote retinol metabolism and lipid metabolism. The target genes of the DE miRNAs were showed high expression in the liver of the neonatal goats after birth and moderately low expression in the liver of the weaned goats (cluster 2), and they were significantly enriched in Metabolism, Human Diseases, and Immune system, such as steroid hormone biosynthesis, metabolism of xenobiotics by cytochrome P450, chemical carcinogenesis, drug metabolism - cytochrome P450, hematopoietic cell lineage, and so on. It is reported that Cytochrome P450s play critical roles in the biosynthesis of steroid hormones. depending on CYP17 activity, the steroid hormone biosynthesis pathway is directed to either the formation of mineralocorticoids and glucocorticoids or sex hormones (Gilep et al., 2011). Furthermore, the pathway with the largest number of enriched genes is pathways in cancer. Studies have shown that miRNAs regulate the malignant behavior of tumor cells (Volinia et al., 2010; Gianpiero and Carlo, 2013), including EMT-related tumor metastasis (Aiello and Kang, 2019). Some miRNAs are intended as potential tumor biomarkers. Therefore, the potential diagnostic, prognostic and therapeutic value of the cancer-associated DE miRNAs in the goat livers in cancers needs further investigations. Therefore, the biological function of the goat liver has been predicted to change with growth and development. The mechanism of action of genes in the goat liver is complex and operates in combination with the multiple RNAs.

In our study, a target regulatory network was constructed with DE miRNAs and target genes from seven GO terms and two KEGG pathways. According to the degree of interaction, the network contained nine core miRNAs. The two KEGG pathways are retinol metabolism and steroid hormone biosynthesis. The pathway of retinol metabolism was in the first cluster, and the pathway of steroid hormone biosynthesis was in the second cluster. Furthermore, they are significantly enriched in the two pathways significantly. In our study, chi-miR-15a-5p has the largest number of target genes, and its targets were significantly enriched in the pathway of Necroptosis. It is reported that miR-15a-5p can silence E2F3 expression, which plays an important role in the Vitamin D3 suppressed cell proliferation. (Li et al., 2020). We predicted that miR-15a-5p may silence gene expression, inhibit the apoptosis pathway, and play an important role in promoting cell proliferation. It is reported that LINC01783 may increase in NSCLC cell lines, and down-regulation of LINC01783 suppressed cell proliferation, migration, and invasion by regulating Notch pathway and sponging miR-432-5p (Deng et al., 2021). Guo et al. found that Notch2 may negatively regulate cell invasion by inhibiting the PI3K-Akt signaling pathway in gastric cancer (Guo et al., 2012). In our study, chi-miR-497-5p and chi-miR-432-5p are all target NOTCH2, and NOTCH2 is significantly enriched in the PI3K-Akt signaling pathway. We predicted that chi-miR-15a-5p, chi-miR-497-5p, and chi-miR-432-5p play a role in cell proliferation, migration, and invasion of goat kids livers. Let-7b-5p can be used as a potential biomarker for drug-induced liver injury (Kagawa et al., 2018). Let-7b-5p could participate in the glycolytic pathway by regulating target genes (Zhou et al., 2018). We find that the targets of chi-Let-7b-5p were enriched in Retinol metabolism. MiR-150 reduced glucose utilization by directly decreasing the expression and translocation of GLUT4 in the cardiomyocytes with insulin resistance (Ju et al., 2020). In our study, the target genes of MiR-150 were not significantly enriched in glucose metabolism-related pathways, which may be affected by related hormones and cytokines in the body. Enhancement of MBNL1-AS1 or inhibition of miR-412-3p was shown to decrease CSC proliferation, migration, and invasion but promote apoptosis (Zhu et al., 2020). In our study, the expression of chi-miR-412-3p was highest at 12 weeks of age in goat liver, because miR-412-3p played different roles in different tissues and cells. MiR-30b-3p overexpression significantly repressed cell viability, proliferation, migration, and invasion of HCC cells *in vitro* (Gao et al., 2019; Zhao et al., 2012). Bufalin inhibits cell proliferation and migration of HCC cells via APOBEC3F induced intestinal immune network for IgA production signaling pathway (Yang et al., 2018). We found that the target genes of chi-miR-30b-3p are significantly enriched in intestinal immune network for IgA production signaling pathway.

In summary, this study sequenced DE miRNAs in the five developmental stages of goat kids livers, and functional enrichment analysis showed that target genes were mainly concentrated in the pathways related to cell proliferation, metabolism, and the occurrence and development of disease. The research provides essential information for studying the livers of goat kids in development, metabolism, and immune. Our results expanded the repertoire of goat miRNA and may be of help in better understanding the mechanism of early development from the perspective of ruminant liver development.

## Data Availability

The original contributions presented in the study are publicly available. This data can be found here: NCBI-SRA database with a BioProject ID of PRJNA770352 and SRA ID of SRP340855.

## References

[B67] Aiello N. M. Kang Y. (2019). Context-Dependent EMT Programs in Cancer Metastasis. J. Exp. Med. 216, 1016–1026. 10.1084/jem.20181827 30975895 PMC6504222

[B1] Ambros V. (2004). The Functions of Animal microRNAs. Nature 431 (7006), 350–355. 10.1038/nature02871 15372042

[B2] Andrada A. D. Cortés C. C. (2004). Changes in the Composition of Sows' Milk between Days 5 to 26 of Lactation. Span J. Agric. Res. (3), 333–336. 10.5424/sjar/2004023-102

[B3] Baldwin R. L. McLeod K. R. Klotz J. L. Heitmann R. N. (2004). Rumen Development, Intestinal Growth and Hepatic Metabolism in the Pre- and Postweaning Ruminant. J. Dairy Sci. 87 (Supplement), E55–E65. 10.3168/jds.S0022-0302(04)70061-2

[B4] Bartel D. P. (2004). MicroRNAs. Cell 116 (2), 281–297. 10.1016/s0092-8674(04)00045-5 14744438

[B6] Buranakarl C. Thammacharoen S. Semsirmboon S. Sutayatram S. Nuntapaitoon M. Dissayabutra T. (2021). Effects of Litter Size and Parity Number on Mammary Secretions Including, Insulin-like Growth Factor-1, Immunoglobulin G and Vitamin A of Black Bengal, Saanen and Their Crossbred Goats in Thailand. Vet. Sci. 8 (6), 95. 10.3390/vetsci8060095 34072801 PMC8229495

[B7] Cock P. J. A. Fields C. J. Goto N. Heuer M. L. Rice P. M. (2010). The Sanger FASTQ File Format for Sequences with Quality Scores, and the Solexa/Illumina FASTQ Variants. Nucleic Acids Res. 38 (6), 1767–1771. 10.1093/nar/gkp1137 20015970 PMC2847217

[B8] Deng Y. Zhang L. Luo R. (2021). LINC01783 Facilitates Cell Proliferation, Migration and Invasion in Non-small Cell Lung Cancer by Targeting miR-432-5p to Activate the Notch Pathway. Cancer Cel Int 21 (1), 234. 10.1186/s12935-021-01912-0 PMC8073972 33902591

[B9] Galvani D. B. Pires C. C. Hübner C. H. Carvalho S. Wommer T. P. (2014). Growth Performance and Carcass Traits of Early-Weaned Lambs as Affected by the Nutritional Regimen of Lactating Ewes. Small Ruminant Res. 120 (Issue 1), 1–5. 10.1016/j.smallrumres.2014.03.008

[B10] Gao D. Zhou Z. Huang H. (2019). miR-30b-3p Inhibits Proliferation and Invasion of Hepatocellular Carcinoma Cells via Suppressing PI3K/Akt Pathway. Front. Genet. 10, 1274. 10.3389/fgene.2019.01274 31921311 PMC6923265

[B66] Gianpiero D. L. Carlo M. C. (2013). miRNA Profiling of Cancer. Cur. Opin. Genet. Dev. 23, 3–11. 10.1016/j.gde.2013.01.004 PMC3632255 23465882

[B11] Gilep A. A. Sushko T. A. Usanov S. A. (2011). At the Crossroads of Steroid Hormone Biosynthesis: the Role, Substrate Specificity and Evolutionary Development of CYP17. Biochim. Biophys. Acta (Bba) - Proteins Proteomics 1814 (1), 200–209. 10.1016/j.bbapap.2010.06.021 20619364

[B12] Girard M. Jacquemin E. Munnich A. Lyonnet S. Henrion-Caude A. (2008). miR-122, a Paradigm for the Role of microRNAs in the Liver. J. Hepatol. 48 (4), 648–656. 10.1016/j.jhep.2008.01.019 18291553

[B13] Greenwood P. L. Hunt A. S. Bell A. W. (2004). Effects of Birth Weight and Postnatal Nutrition on Neonatal Sheep: IV. Organ Growth12. J. Anim. Sci. 82 (Issue 2), 422–428. 10.2527/2002.80112850x 14974539

[B14] Guo L.-Y. Li Y. M. Qiao L. Liu T. Du Y. Y. Zhang J. Q. (2012). Notch2 Regulates Matrix Metallopeptidase 9viaPI3K/AKT Signaling in Human Gastric Carcinoma Cell MKN-45. Wjg 18 (48), 7262–7270. 10.3748/wjg.v18.i48.7262 23326131 PMC3544028

[B15] He L. Hannon G. J. (2004). MicroRNAs: Small RNAs with a Big Role in Gene Regulation. Nat. Rev. Genet. 5 (7), 522–531. 10.1038/nrg1379 15211354

[B16] He S. Wang H. Liu R. He M. Che T. Jin L. (2017). mRNA N6-Methyladenosine Methylation of Postnatal Liver Development in Pig. PLoS One 12 (3), e0173421. 10.1371/journal.pone.0173421 28267806 PMC5340393

[B17] He Y. Huang C. Zhang S.-p. Sun X. Long X.-r. Li J. (2012). The Potential of microRNAs in Liver Fibrosis. Cell Signal. 24 (12), 2268–2272. 10.1016/j.cellsig.2012.07.023 22884954

[B18] Hoffmann T. W. Gilles D. Abderrahmane B. (2012). MicroRNAs and Hepatitis C Virus: toward the End of miR-122 Supremacy. Virol. J. 9, 109. 10.1186/1743-422X-9-109 22691570 PMC3489824

[B19] Jahn S. C. Gay L. A. Weaver C. J. Renne R. Langaee T. Y. Stacpoole P. W. (2020). Age-Related Changes in miRNA Expression Influence GSTZ1 and Other Drug Metabolizing Enzymes. Drug Metab. Dispos 48 (7), 563–569. 10.1124/dmd.120.090639 32357971 PMC7289049

[B20] Ju J. Xiao D. Shen N. Zhou T. Che H. Li X. (2020). miR-150 Regulates Glucose Utilization through Targeting GLUT4 in Insulin-Resistant Cardiomyocytes. Acta Biochim. Biophys. Sin (Shanghai) 52 (10), 1111–1119. 10.1093/abbs/gmaa094 33085741

[B21] Kagawa T. Shirai Y. Oda S. Yokoi T. (2018). Identification of Specific MicroRNA Biomarkers in Early Stages of Hepatocellular Injury, Cholestasis, and Steatosis in Rats. Toxicol. Sci. 166 (1), 228–239. 10.1093/toxsci/kfy200 30125006

[B22] Kang H. Heo S. Shin J. J. Ji E. Tak H. Ahn S. (2019). A miR‐194/PTBP1/CCND3 axis Regulates Tumor Growth in Human Hepatocellular Carcinoma. J. Pathol. 249 (3), 395–408. 10.1002/path.5325 31301177

[B23] Kim V. N. Han J. Siomi M. C. (2009). Biogenesis of Small RNAs in Animals. Nat. Rev. Mol. Cel Biol 10 (2), 126–139. 10.1038/nrm2632 19165215

[B24] Kiss T. (2002). Small Nucleolar RNAs. Cell 109 (2), 145–148. 10.1016/s0092-8674(02)00718-3 12007400

[B25] Kunden R. D. Khan J. Q. Ghezelbash S. Wilson J. A. (2020). The Role of the Liver-specific microRNA, miRNA-122 in the HCV Replication Cycle. Ijms 21 (16), 5677. 10.3390/ijms21165677 PMC7460827 32784807

[B26] Li L. He M. L. Wang K. Zhang Y. S. (2018). Buffering Agent via Insulin-Mediated Activation of PI3K/AKT Signaling Pathway to Regulate Lipid Metabolism in Lactating Goats. Physiol. Res. 67 (5), 753–764. 10.33549/physiolres.933698 30044118

[B27] Li Y. Lin Q. Chang S. e. Zhang R. Wang J. (2020). Vitamin D3 Mediates miR-15a-5p I-nhibition of L-iver C-ancer C-ell P-roliferation via T-argeting E2F3. Oncol. Lett. 20 (1), 292–298. 10.3892/ol.2020.11572 PMC7285896 32565955

[B28] Li Z.-H. Wang Y.-F. He D.-D. Zhang X.-M. Zhou Y.-L. Yue H. (2019). Let-7f-5p Suppresses Th17 Differentiation via Targeting STAT3 in Multiple Sclerosis. Aging 11 (13), 4463–4477. 10.18632/aging.102093 31326963 PMC6660039

[B29] Liang G. Malmuthuge N. McFadden T. B. Bao H. Griebel P. J. Stothard P. (2014). Potential Regulatory Role of microRNAs in the Development of Bovine Gastrointestinal Tract during Early Life. PLoS One 9 (3), e92592. 10.1371/journal.pone.0092592 24682221 PMC3969364

[B30] Lima J. P. (1980). Anatomy and Physiology of the Liver Secretory Apparatus. Arq Gastroenterol. 17 (3), 149–160. 7016087

[B31] Liu Y. Jin L. Lou P. Gu Y. Li M. Li X. (2017). Dynamic microRNAome Profiles in the Developing Porcine Liver. Biosci. Biotechnol. Biochem. 81 (1), 127–134. 10.1080/09168451.2016.1240602 27702394

[B32] Livak K. J. Schmittgen T. D. (2001). Analysis of Relative Gene Expression Data Using Real-Time Quantitative PCR and the 2−ΔΔCT Method. Methods 25, 402–408. 10.1006/meth.2001.1262 11846609

[B33] Love M. I. Huber W. Anders S. (2014). Moderated Estimation of Fold Change and Dispersion for RNA-Seq Data with DESeq2. Genome Biol. 15, 550. 10.1186/s13059-014-0550-8 25516281 PMC4302049

[B34] Lv X. Liang J. Wang Z. (2020). MiR-21-5p Reduces Apoptosis and Inflammation in Rats with Spinal Cord Injury through PI3K/AKT Pathway. Panminerva Med. 2020. 10.23736/S0031-0808.20.03974-9 32720795

[B35] Miranda R. C. Pietrzykowski A. Z. Tang Y. Sathyan P. Mayfield D. Keshavarzian A. (2010). MicroRNAs: Master Regulators of Ethanol Abuse and Toxicity? Alcohol. Clin. Exp. Res. 34 (4), 575–587. 10.1111/j.1530-0277.2009.01126.x 20102566 PMC2925252

[B36] Momen-Heravi F. Saha B. Kodys K. Catalano D. Satishchandran A. Szabo G. (2015). Increased Number of Circulating Exosomes and Their microRNA Cargos Are Potential Novel Biomarkers in Alcoholic Hepatitis. J. Transl Med. 13, 261. 10.1186/s12967-015-0623-9 26264599 PMC4533956

[B37] Nelson P. Kiriakidou M. Sharma A. Maniataki E. Mourelatos Z. (2003). The microRNA World: Small Is Mighty. Trends Biochem. Sci. 28 (10), 534–540. 10.1016/j.tibs.2003.08.005 14559182

[B38] Otsuka M. Kishikawa T. Yoshikawa T. Yamagami M. Ohno M. Takata A. (2017). MicroRNAs and Liver Disease. J. Hum. Genet. 62 (1), 75–80. 10.1038/jhg.2016.53 27225852

[B39] Prosser C. G. (2021). Compositional and Functional Characteristics of Goat Milk and Relevance as a Base for Infant Formula. J. Food Sci. 86 (2), 257–265. 10.1111/1750-3841.15574 33438254

[B40] Ran R.-Z. Chen J. Cui L.-J. Lin X.-L. Fan M.-M. Cong Z.-Z. (2019). miR-194 Inhibits Liver Cancer Stem Cell Expansion by Regulating RAC1 Pathway. Exp. Cel Res. 378 (1), 66–75. 10.1016/j.yexcr.2019.03.007 30844391

[B41] Reinke H. Asher G. (2016). Circadian Clock Control of Liver Metabolic Functions. Gastroenterology 150 (3), 574–580. 10.1053/j.gastro.2015.11.043 26657326

[B42] Santos A. Giráldez F. J. Trevisi E. Lucini L. Frutos J. Andrés S. (2018). Liver Transcriptomic and Plasma Metabolomic Profiles of Fattening Lambs Are Modified by Feed Restriction during the Suckling Period1. J. Anim. Sci. 96 (4), 1495–1507. 10.1093/jas/sky029 29471523 PMC6140908

[B43] Sengupta D. Cassel T. Teng K.-y. Aljuhani M. Chowdhary V. K. Hu P. (2020). Regulation of Hepatic Glutamine Metabolism by miR-122. Mol. Metab. 34, 174–186. 10.1016/j.molmet.2020.01.003 32180557 PMC7044666

[B44] Si-Tayeb K. Lemaigre F. P. Duncan S. A. (2010). Organogenesis and Development of the Liver. Develop. Cel 18 (2), 175–189. 10.1016/j.devcel.2010.01.011 20159590

[B45] Swanson K. S. Merchen N. R. Erdman J. W. Jr Drackley J. K. Orias F. Morin D. E. (2000). Influence of Dietary Vitamin A Content on Serum and Liver Vitamin A Concentrations and Health in Preruminant Holstein Calves Fed Milk Replacer,. J. Dairy Sci. 83 (9), 2027–2036. 10.3168/jds.S0022-0302(00)75083-1 11003235

[B46] Taganov K. D. Boldin M. P. Baltimore D. (2007). MicroRNAs and Immunity: Tiny Players in a Big Field. Immunity 26 (2), 133–137. 10.1016/j.immuni.2007.02.005 17307699

[B47] Tan W. Gu Z. Leng J. Zou x. Chen H. Min F. (2019). Let-7f-5p Ameliorates Inflammation by Targeting NLRP3 in Bone Marrow-Derived Mesenchymal Stem Cells in Patients with Systemic Lupus Erythematosus. Biomed. Pharmacother. 118, 109313. 10.1016/j.biopha.2019.109313 31545233

[B48] Tian J. Rui Y. J. Xu Y. J. Zhang S. A. (2018). MiR-143-3p Regulates Early Cartilage Differentiation of BMSCs and Promotes Cartilage Damage Repair through Targeting BMPR2. Eur. Rev. Med. Pharmacol. Sci. 22 (24), 8814–8821. 10.26355/eurrev_201812_16649 30575923

[B49] Tzur G. Israel A. Levy A. Benjamin H. Meiri E. Shufaro Y. (2009). Comprehensive Gene and microRNA Expression Profiling Reveals a Role for microRNAs in Human Liver Development. PLoS One 4 (10), e7511. 10.1371/journal.pone.0007511 19841744 PMC2760133

[B50] Valdmanis P. N. Gu S. Chu K. Jin L. Zhang F. Munding E. M. (2016). RNA Interference-Induced Hepatotoxicity Results from Loss of the First Synthesized Isoform of microRNA-122 in Mice. Nat. Med. 22 (5), 557–562. 10.1038/nm.4079 27064447 PMC4860119

[B64] van den Borne J. J. Lobley G. E. Verstegen M. W. Muijlaert J. M. Alferink S. J. Gerrits W. J. (2007). Body Fat Deposition Does Not Originate From Carbohydrates in Milk-Fed Calves. J. Nutrition 137 (10), 2234–2241. 10.1093/jn/137.10.2234 17885004

[B51] Voinnet O. (2005). Induction and Suppression of RNA Silencing: Insights from Viral Infections. Nat. Rev. Genet. 6 (3), 206–220. 10.1038/nrg1555 15703763

[B65] Volinia S. Galasso M. Costinean S. Tagliavini L. Gamberoni G. Drusco A. (2010). Reprogramming of Mirna Networks in Cancer And Leukemia. Genome Res. 20, 589–599. 10.1101/gr.098046.109 20439436 PMC2860161

[B52] Wang B. Zhang A. Wang H. Klein J. D. Tan L. Wang Z.-M. (2019). miR-26a Limits Muscle Wasting and Cardiac Fibrosis through Exosome-Mediated microRNA Transfer in Chronic Kidney Disease. Theranostics 9 (7), 1864–1877. 10.7150/thno.29579 31037144 PMC6485283

[B53] Wang H. Luo J. Zhang T. Tian H. Ma Y. Xu H. (2016). MicroRNA-26a/b and Their Host Genes Synergistically Regulate Triacylglycerol Synthesis by Targeting theINSIG1gene. RNA Biol. 13 (5), 500–510. 10.1080/15476286.2016.1164365 27002347 PMC4962806

[B54] Xing X. Guo S. Zhang G. Liu Y. Bi S. Wang X. (2020). miR-26a-5p Protects against Myocardial Ischemia/reperfusion Injury by Regulating the PTEN/PI3K/AKT Signaling Pathway. Braz. J. Med. Biol. Res. 53 (2), e9106. 10.1590/1414-431X20199106 31994603 PMC6984371

[B55] Yang Z. Tao Y. Xu X. Cai F. Yu Y. Ma L. (2018). Bufalin Inhibits Cell Proliferation and Migration of Hepatocellular Carcinoma Cells via APOBEC3F Induced Intestinal Immune Network for IgA Production Signaling Pathway. Biochem. Biophysical Res. Commun. 503 (3), 2124–2131. 10.1016/j.bbrc.2018.07.169 30100060

[B56] Yi P.-S. Zhang M. Xu M. Q. (2016). Role of microRNA in Liver Regeneration. Hepatobiliary Pancreat. Dis. Int. 15 (2), 141–146. 10.1016/s1499-3872(15)60036-4 27020629

[B57] Yin C. Evason K. J. Asahina K. Stainier D. Y. R. (2013). Hepatic Stellate Cells in Liver Development, Regeneration, and Cancer. J. Clin. Invest. 123 (5), 1902–1910. 10.1172/JCI66369 23635788 PMC3635734

[B62] Yin S. Fan Y. Zhang H. Zhao Z. Hao Y. Li J. (2016). Differential Tgfβ Pathway Targeting By Mir-122 In Humans And Mice Affects Liver Cancer Metastasis. Nat. Commun. 7, 11012. 10.1038/ncomms11012 26987776 PMC4802055

[B63] Zaret K. S. (2002). Regulatory Phases Of Early Liver Development: Paradigms Of Organogenesis. Nat. Rev. Genet. 3 (7), 499–512. 10.1038/nrg837 12094228

[B58] Zhang Z. Tan X. Luo J. Yao H. Si Z. Tong J-S. (2020). The miR-30a-5p/CLCF1 axis Regulates Sorafenib Resistance and Aerobic Glycolysis in Hepatocellular Carcinoma. Cell Death Dis 11 (10), 902. 10.1038/s41419-020-03123-3 33097691 PMC7584607

[B59] Zhao X. Yang Z. Li G. Li D. Zhao Y. Wu Y. (2012). The Role and Clinical Implications of microRNAs in Hepatocellular Carcinoma. Sci. China Life Sci. 55 (10), 906–919. 10.1007/s11427-012-4384-x 23108868

[B60] Zhou R. Zhang Y. Du G. Han L. Zheng S. Liang J. (2018). Down-regulated Let-7b-5p Represses Glycolysis Metabolism by Targeting AURKB in Asthenozoospermia. Gene 663, 83–87. 10.1016/j.gene.2018.04.022 29653228

[B61] Zhu K. Wang Y. Liu L. Li S. Yu W. (2020). Long Non-coding RNA MBNL1-AS1 Regulates Proliferation, Migration, and Invasion of Cancer Stem Cells in colon Cancer by Interacting with MYL9 via Sponging microRNA-412-3p. Clin. Res. Hepatol. Gastroenterol. 44 (1), 101–114. 10.1016/j.clinre.2019.05.001 31255531

