# Supplementary material for: Characterization of the microRNA Expression Profiles in the Goat Kid Liver
Source: Front Genet. 2022 Jan 10;12:794157. doi: 10.3389/fgene.2021.794157 (PMC8784682; doi:10.3389/fgene.2021.794157)
Supplement: Supplementary file 1 [file Table1.DOCX]

| **ID** | **Body Length (cm)** | **Body Height (cm)** | **Body Weight (kg)** |
| --- | --- | --- | --- |
| **D1-1** | 33.12 | 34.67 | 3.02 |
| **D1-2** | 33.53 | 35.23 | 3.45 |
| **D1-3** | 32.85 | 34.64 | 2.85 |
| **D1-4** | 33.53 | 35.36 | 3.15 |
| **D1-5** | 33.76 | 35.73 | 3.39 |
| **W2-1** | 36.24 | 40.25 | 4.85 |
| **W2-2** | 37.75 | 39.68 | 4.75 |
| **W2-3** | 36.53 | 39.72 | 4.63 |
| **W2-4** | 37.53 | 40.55 | 5.12 |
| **W2-5** | 37.75 | 39.59 | 4.95 |
| **W4-1** | 39.23 | 32.36 | 4.15 |
| **W4-2** | 39.64 | 32.48 | 4.34 |
| **W4-3** | 40.28 | 32.94 | 4.47 |
| **W4-4** | 39.54 | 33.57 | 4.75 |
| **W4-5** | 40.53 | 33.85 | 5.25 |
| **W8-1** | 47.55 | 42.24 | 7.55 |
| **W8-2** | 47.26 | 42.83 | 7.22 |
| **W8-3** | 46.94 | 43.36 | 7.29 |
| **W8-4** | 47.39 | 42.57 | 7.24 |
| **W8-5** | 47.58 | 42.74 | 7.45 |
| **W12-1** | 51.53 | 47.95 | 11.25 |
| **W12-2** | 50.97 | 47.58 | 10.45 |
| **W12-3** | 50.43 | 47.24 | 10.27 |
| **W12-4** | 51.26 | 47.93 | 10.93 |
| **W12-5** | 51.37 | 48.25 | 11.65 |

**Table S1 Body size information of Laiwu black goat for microRNA profile analysis**
