# Supplementary material for: Characterization of the microRNA Expression Profiles in the Goat Kid Liver
Source: Front Genet. 2022 Jan 10;12:794157. doi: 10.3389/fgene.2021.794157 (PMC8784682; doi:10.3389/fgene.2021.794157)
Supplement: Supplementary file 3 [file Table2.DOCX]

**Table S2 The qRT-PCR primers used for validation**

| **ID** | **primer sequence** | **Base number** |
| --- | --- | --- |
| **chi-let-7b-5p** | UGAGGUAGUAGGUUGUGUGGUU | 22 |
| **chi-miR-103-3p** | AGCAGCAUUGUACAGGGCUAUGA | 22 |
| **chi-miR-133a-3p** | UUUGGUCCCCUUCAACCAGCUGU | 22 |
| **chi-miR-15a-5p** | UAGCAGCACAUAAUGGUUUGUGG | 22 |
| **chi-miR-16a-5p** | UAGCAGCACGUAAAUAUUGGAG | 22 |
| **chi-miR-221-3p** | AGCUACAUUGUCUGCUGGGUUU | 22 |
| **chi-miR-335-5p** | CUUUUUGCGGUCUGGGCUUGC | 21 |
| **chi-miR-340-5p** | AUCUCAGGUUCGUCAGCCCGCA | 22 |
| **chi-miR-3431-5p** | UAGCAGCACAGAAAUGUUGG | 21 |
| **chi-miR-369-3p** | CUGGGAGGUGGAUGUUUACUUC | 22 |
| **chi-miR-379-5p** | CUCACUGAUCAAUGAAUGCAA | 21 |
| **chi-miR-411a-5p** | AGGCAGUGUAAUUAGCUGAUUGU | 22 |
| **chi-miR-424-5p** | CGGGGUUUUGAGGGCGAGAUGA | 22 |
| **chi-miR-450-5p** | AGGGGUGCUAUCUGUGGUUGAGG | 22 |
| **chi-miR-487b-5p** | AUGGUUGACCACAGAACAUGCGC | 22 |
