# Supplementary material for: Characterization of the microRNA Expression Profiles in the Goat Kid Liver
Source: Front Genet. 2022 Jan 10;12:794157. doi: 10.3389/fgene.2021.794157 (PMC8784682; doi:10.3389/fgene.2021.794157)
Supplement: Supplementary file 4 [file Table5.DOCX]

**Table S5 Summary of sequencing read alignment to the goat reference genome**

| **Sample** | **Total reads** | **Total mapped** | **Mapped reads (+)** | | **Mapped reads (-)** | |
| --- | --- | --- | --- | --- | --- | --- |
| **D1_1** | 11355718 | 10581231 | 5921093 | 5596689 | |  |
| **D1_2** | 11716320 | 10958699 | 6055228 | 5952429 | |  |
| **D1_3** | 9057602 | 8077982 | 4718662 | 4189848 | |  |
| **D1_4** | 11806062 | 10824161 | 5867865 | 5879987 | |  |
| **D1_5** | 9166912 | 8576988 | 4674367 | 4668966 | |  |
| **W2_1** | 10882336 | 10074026 | 5898305 | 5158295 | |  |
| **W2_2** | 13008993 | 12366584 | 6862539 | 6654519 | |  |
| **W2_3** | 10514997 | 9682176 | 5191462 | 5319020 | |  |
| **W2_4** | 11821957 | 10959381 | 5942059 | 6037604 | |  |
| **W2_5** | 9361606 | 8578006 | 4679836 | 4729619 | |  |
| **W4_1** | 11600516 | 10909415 | 6071533 | 5953788 | |  |
| **W4_2** | 9997430 | 9236667 | 4145771 | 5856487 | |  |
| **W4_3** | 10515719 | 9721257 | 5762844 | 4997994 | |  |
| **W4_4** | 11762793 | 10846348 | 6441080 | 5559617 | |  |
| **W4_5** | 9746074 | 9251867 | 5300370 | 4836568 | |  |
| **W8_1** | 9870972 | 9314759 | 5062004 | 5141150 | |  |
| **W8_2** | 10117181 | 9272753 | 5166662 | 4985850 | |  |
| **W8_3** | 11354316 | 10519635 | 6030526 | 5577939 | |  |
| **W8_4** | 12751848 | 11923959 | 6664333 | 6496454 | |  |
| **W8_5** | 12417867 | 11852533 | 6872835 | 6163201 | |  |
| **W12_1** | 12130802 | 11184576 | 6351332 | 5984239 | |  |
| **W12_2** | 14498342 | 13309642 | 7830212 | 6885196 | |  |
| **W12_3** | 10629630 | 9913121 | 5548445 | 5418838 | |  |
| **W12_4** | 11193451 | 10546182 | 5902935 | 5706593 | |  |
| **W12_5** | 10277634 | 9617041 | 5048553 | 5569021 | |  |
