# Supplementary material for: Characterization of the microRNA Expression Profiles in the Goat Kid Liver
Source: Front Genet. 2022 Jan 10;12:794157. doi: 10.3389/fgene.2021.794157 (PMC8784682; doi:10.3389/fgene.2021.794157)
Supplement: Supplementary file 5 [file Table3.DOCX]

**Table S3 clean reads filtering results**

| **Sample** | | **Raw Reads** | **Clean Reads** | **Error rate (%)** | **Q20(%)** | **Q30(%)** | **Useful reads(18-32nt)** |
| --- | --- | --- | --- | --- | --- | --- | --- |
| **D1_1** | 12493978 | 12136272 | 0.0228 | 98.93 | 96.42 | 11355718 |  |
| **D1_2** | 12695777 | 12358104 | 0.0227 | 98.95 | 96.49 | 11716320 |  |
| **D1_3** | 10129894 | 9794740 | 0.0229 | 98.83 | 96.31 | 9057602 |  |
| **D1_4** | 13132597 | 12731657 | 0.0237 | 98.4 | 95.59 | 11806062 |  |
| **D1_5** | 10017703 | 9741230 | 0.0233 | 98.58 | 95.98 | 9166912 |  |
| **W2_1** | 11811273 | 11493856 | 0.0227 | 98.94 | 96.59 | 10882336 |  |
| **W2_2** | 13721955 | 13485022 | 0.0225 | 99.05 | 96.76 | 13008993 |  |
| **W2_3** | 11591588 | 11230791 | 0.0229 | 98.85 | 96.35 | 10514997 |  |
| **W2_4** | 12792079 | 12501215 | 0.0228 | 98.88 | 96.41 | 11821957 |  |
| **W2_5** | 10427297 | 10016750 | 0.0228 | 98.94 | 96.43 | 9361606 |  |
| **W4_1** | 12374534 | 12102743 | 0.0229 | 98.88 | 96.37 | 11600516 |  |
| **W4_2** | 10876291 | 10644223 | 0.0236 | 98.48 | 95.65 | 9997430 |  |
| **W4_3** | 11831046 | 11314194 | 0.0227 | 98.94 | 96.51 | 10515719 |  |
| **W4_4** | 12864274 | 12484295 | 0.0229 | 98.82 | 96.46 | 11762793 |  |
| **W4_5** | 10297494 | 10115991 | 0.0229 | 98.8 | 96.35 | 9746074 |  |
| **W8_1** | 10486310 | 10315472 | 0.0226 | 98.96 | 96.64 | 9870972 |  |
| **W8_2** | 11011901 | 10755935 | 0.0228 | 98.95 | 96.39 | 10117181 |  |
| **W8_3** | 12151000 | 11985174 | 0.0229 | 98.84 | 96.3 | 11354316 |  |
| **W8_4** | 13562884 | 13377710 | 0.0232 | 98.65 | 96.18 | 12751848 |  |
| **W8_5** | 13097861 | 12911744 | 0.0227 | 98.9 | 96.56 | 12417867 |  |
| **W12_1** | 13026424 | 12797556 | 0.0231 | 98.7 | 96.24 | 12130802 |  |
| **W12_2** | 15692244 | 15428435 | 0.0225 | 99.02 | 96.76 | 14498342 |  |
| **W12_3** | 11613024 | 11355761 | 0.0226 | 99.02 | 96.68 | 10629630 |  |
| **W12_4** | 12034612 | 11788022 | 0.0227 | 98.98 | 96.53 | 11193451 |  |
| **W12_5** | 10757996 | 10632218 | 0.0236 | 98.61 | 95.48 | 10277634 |  |
