# Supplementary material for: Characterization of the microRNA Expression Profiles in the Goat Kid Liver
Source: Front Genet. 2022 Jan 10;12:794157. doi: 10.3389/fgene.2021.794157 (PMC8784682; doi:10.3389/fgene.2021.794157)
Supplement: Supplementary file 6 [file Table4.DOCX]

**Table S4 ncRNA and repeat sequence annotation.**

| **Sample ID** | **rRNA** | **tRNA** | **snoRNA** | **snRNA** | **Repeat reads** | **Unannotated** | | **Total** |
| --- | --- | --- | --- | --- | --- | --- | --- | --- |
| D1 | 473205±51063 | 85894±6064 | 4942± 229 | 5978±726 | 115629±6986 | 379306±25013 | 10620522±620584 | |
| W2 | 386091±35854 | 173639±26201 | 4237±405 | 6333±928 | 112957± 6591 | 326533±14109 | 11117977±615446 | |
| W4 | 352518±52448 | 168424±28755 | 4070±323 | 6403±766 | 101953±11829 | 336241±26165 | 10724506±410791 | |
| W8 | 372772±50622 | 111296±32393 | 4029±215 | 4364±301 | 88631±4705 | 335599±26400 | 11302436±583163 | |
| W12 | 399296±68228 | 185957±38816 | 4241±317 | 5497±957 | 87590±9808 | 361631±30833 | 11745971±756014 | |
